# Psoriatic Arthritis: From Diagnosis to Treatment

**DOI:** 10.3390/jcm14228151

**Published:** 2025-11-17

**Authors:** Renuka Kannappan, Sarah Kim, Arthur Lau, Lawrence H. Brent

**Affiliations:** 1 Department of Internal Medicine, Temple University Hospital, Philadelphia, PA 19140, USA; sarah.kim@tuhs.temple.edu; 2 Department of Rheumatology, Jefferson-Einstein Hospital, Philadelphia, PA 19141, USA; arthur.lau@jefferson.edu; 3 Department of Rheumatology, Temple University Hospital, Philadelphia, PA 19140, USA; lawrence.brent@tuhs.temple.edu

**Keywords:** psoriasis, psoriatic arthritis, imaging, ultrasound, DMARDs, prevention

## Abstract

Psoriatic arthritis (PsA) is a chronic, immune-mediated inflammatory arthritis associated with psoriasis, affecting joints, entheses, and the axial skeleton. While primary care providers and dermatologists frequently encounter psoriasis (PsO), early recognition of PsA remains critical to preventing irreversible joint damage. This paper is written to provide a comprehensive overview of PsA, beginning with a clinical case that highlights diagnostic and therapeutic challenges. In this review, the epidemiology of PsA will be discussed, emphasizing its prevalence and risk factors among patients with PsO. The discussion extends to the underlying pathogenesis, focusing on genetic predisposition, environmental triggers, and key cytokines, including TNF-α, IL-17, and IL-23, that have become targets for advanced therapeutics. The clinical features of PsA are explored in detail, including peripheral and axial arthritis, enthesitis, dactylitis, and extra-articular manifestations. Diagnostic approaches are discussed, with a focus on the Classification Criteria for Psoriatic Arthritis (CASPAR) and Moll & Wright criteria. Additionally, we examine screening tools designed to facilitate early detection in dermatology clinics. Diagnostic modalities, including imaging and serologic markers, are reviewed. Finally, we explore the evolving landscape of PsA treatment, spanning conventional synthetic disease-modifying antirheumatic drugs (csDMARDs), biologic agents (bDMARDs), and targeted synthetic DMARDs (tsDMARDs). Given the increasing availability of cytokine-targeted therapies, an interdisciplinary approach between dermatologists and rheumatologists is essential for optimizing outcomes in PsA patients. Patients with PsA are cared for by rheumatologists, dermatologists, and primary care providers who help manage the comorbidities associated with PsA. By bridging primary care, dermatology, and rheumatology in the care of PsA, this paper aims to enhance understanding of PsA for facilitating early identification and timely intervention for improved patient care.

## 1. Introduction

PsO is an immune-mediated disease characterized by erythematous and scaly plaques predominantly found on the skin; however, it also frequently affects the scalp and nails [1]. Up to 30% of patients with PsO may develop psoriatic arthritis (PsA) [2]. PsA is a chronic, progressive, inflammatory arthritis that develops from a combination of genetic and environmental factors that trigger inflammation. [3]. The clinical course of the disease is highly variable, ranging from mild, nondestructive disease to progressive disease involving various domains such as arthritis, spondylitis, enthesitis, and dactylitis [4]. Chronic inflammation may lead to permanent joint destruction and disability that can be prevented with early detection and treatment [5]. The development of biologics and targeted therapies has significantly improved both short-term and long-term outcomes, including reductions in skin and musculoskeletal manifestations of PsA [6]. Dermatologists and primary care physicians are crucial in screening for PsA, early detection, and promoting referrals to specialists such as rheumatologists [1]. This review article covers evidence on the epidemiology, pathogenesis, diagnosis, screening, and management of PsA. This review is valuable in that it includes several methods that can be used by primary care providers and dermatologists to screen for possible PsA in patients with psoriasis and potential biomarkers, as well as possible prevention of PsA and potential new therapies.

## 2. Clinical Case

**Figure 1:**
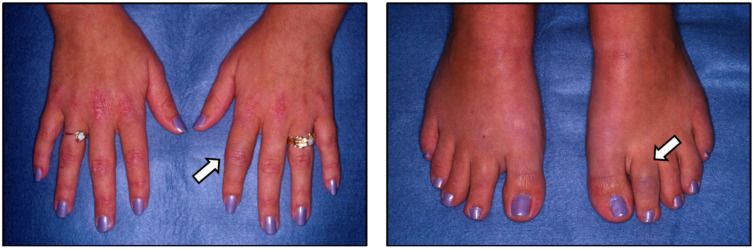
PsA with asymmetric oligoarticular arthritis. White arrows demonstrate dactylitis.

**Figure 2:**
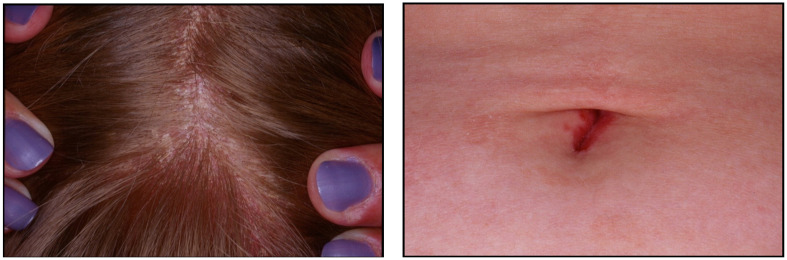
PsO in the scalp and umbilicus.

We present a case of a 29-year-old female who presented with 3 months of pain and swelling in several joints of her hands and feet. She noted 45 min of morning stiffness. She had been taking over-the-counter naproxen for her symptoms with minimal relief. She denied other connective tissue, eye, or gastrointestinal symptoms. Musculoskeletal exam showed synovitis of several proximal interphalangeal joints in the hands and interphalangeal joints in the feet, as seen in Figure 1. Other joints, including the spine, were normal. On careful skin exam, erythematous scaly lesions were seen in the scalp and umbilicus, as seen in Figure 2. Laboratory studies showed complete blood count and comprehensive metabolic panel within normal limits. ESR was elevated at 47 mm/h, and CRP was elevated at 15.7 mg/L. RF and CCP were negative; no HLA-B27 testing was performed. Plain radiographs of the hands and feet were normal except for soft tissue swelling. Negative RF and CCP, along with normal plain radiographs, helped differentiate this patient’s condition from rheumatoid arthritis. The patient was diagnosed with PsA, was treated with methotrexate, and had complete resolution of her joint and skin manifestations. After 6 months, she moved out of state and was lost to follow-up. This case demonstrates classic symptoms and physical exam findings, such as dactylitis and psoriasis plaques, that are seen in patients who present with PsA.

## 3. Epidemiology

The prevalence of PsO is about 2% in the general population [7]. In contrast, the prevalence of PsA in the general population around the world ranges from 0.1% to 1% [8,9]. In North America, the prevalence of PsA in patients with PsO is 19.5% [10]. Additionally, PsO likely occurs before PsA in 17% of patients [11]. The risk of PsA in patients with PsO increases over time; the prevalence of PsA increases to 20.5% over 30 years in patients with PsO [12]. The annual incidence rate of PsA in patients with PsO was 1.87 [13].

The presence of severe skin disease, nail disease, and uveitis in patients with PsO has been associated with the development of PsA [14,15]. Additionally, physical trauma in patients with PsO has been associated with the development of PsA, as well as lifting heavy loads and infections requiring antibiotic therapy [15,16]. In contrast, treatment of patients with PsO with biologic DMARDs has been associated with reduced incidence of PsA compared to those treated with methotrexate [17]. Although PsO is associated with elevated mortality risk, the data regarding mortality risk in patients with PsA have been conflicting. A recent nationwide population-based cohort study in Sweden suggests that comorbidities, such as cancer and cardiovascular disease, drive the increased mortality risk in patients with PsA [18].

## 4. Etiology and Pathogenesis

Genetics and environmental triggers both contribute to the development of PsA. The genetics of PsA is complicated, and some genes are associated with PsA only, others with PsO only, and some with both PsA and PsO [19]. The HLA-B*0801, HLA-B*3901, and HLA-B*2705 genes are associated with PsA but not PsO [20]. In contrast, the HLA-C*0602 gene is much more strongly associated with PsO than PsA [19]. Additionally, different HLA genes are associated with specific phenotypes in PsA and PsO, characteristic immune cell activation, and distinct production of proinflammatory cytokines [21,22]. The HLA-B*0801, HLA-B*38, HLA-B*3901, and HLA-C*0701 genes are associated with peripheral arthritis and spondylitis with asymmetric sacroiliitis, discontinuous spondylitis with non-marginal syndesmophytes (psoriatic spondylitis), while the HLA-B*27-5 gene is associated with symmetric sacroiliitis, gradually ascending spondylitis, and marginal syndesmophytes (typical of ankylosing spondylitis) and enthesitis [22,23,24].

**Figure 3:**
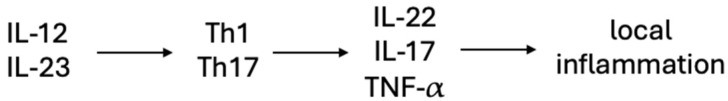
Proposed immunologic pathway of PsA.

Non-MHC loci associated with psoriatic arthritis have also been identified and include *IL23R* (IL-23 receptor involved in the IL-17 pathway) and *TRAF3IP2* (TNF-α-induced protein 3 involved in TNF-α-induced inflammation) [25]. One proposal for the pathogenesis of psoriatic arthritis is activation of innate cells in the skin, entheses, and gastrointestinal tract, leading to the production of IL-12 and IL-23. As a result, T cells differentiate into Th1 and Th17 helper T cells, giving rise to IL-22 and IL-17 secretion and ultimately TNF-α secretion. These cytokines, in turn, promote local inflammation, causing cartilage breakdown, bone remodeling, and joint damage [20,26]. Therefore, IL-23, IL-17, and TNF-α are the major targets for many pharmacologic therapies (Figure 3).

## 5. Clinical Features

**Figure 4:**
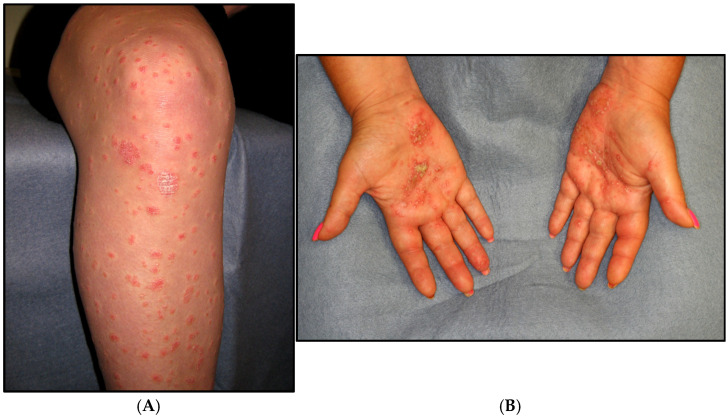
Skin rash of PsO (**A**), plaque PsO (**B**), pustular PsO.

**Figure 5:**
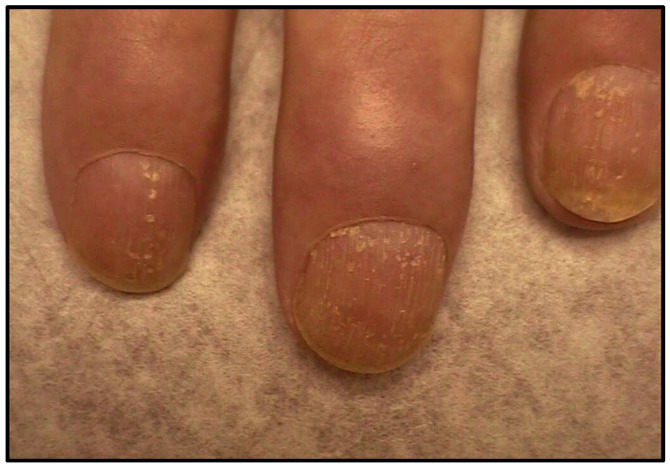
Nail pitting of PsO.

**Figure 6:**
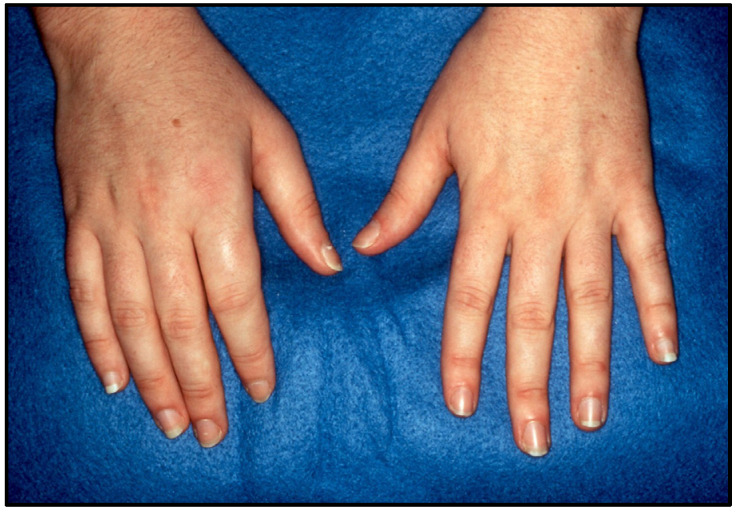
Oligoarticular PsA with asymmetric pattern.

**Figure 7:**
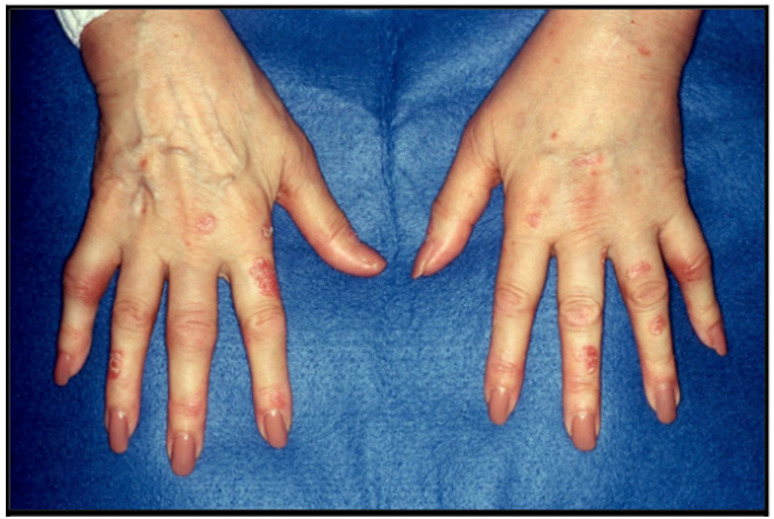
Polyarticular PsA with symmetric pattern.

**Figure 8:**
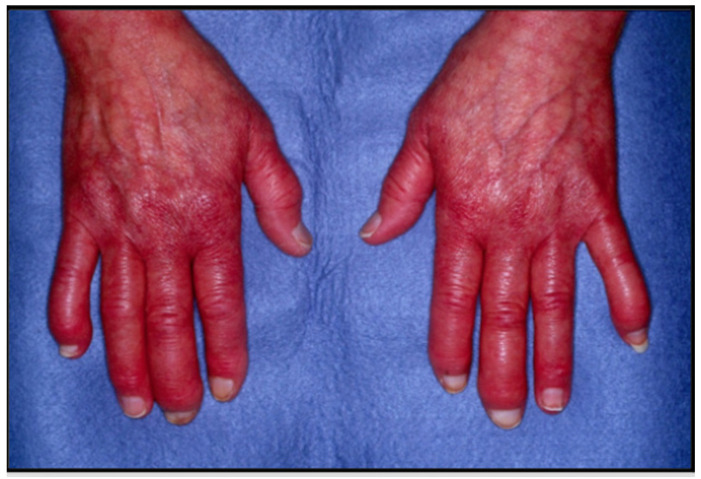
Distal PsA with disease manifestation predominantly in distal interphalangeal joints.

**Figure 9:**
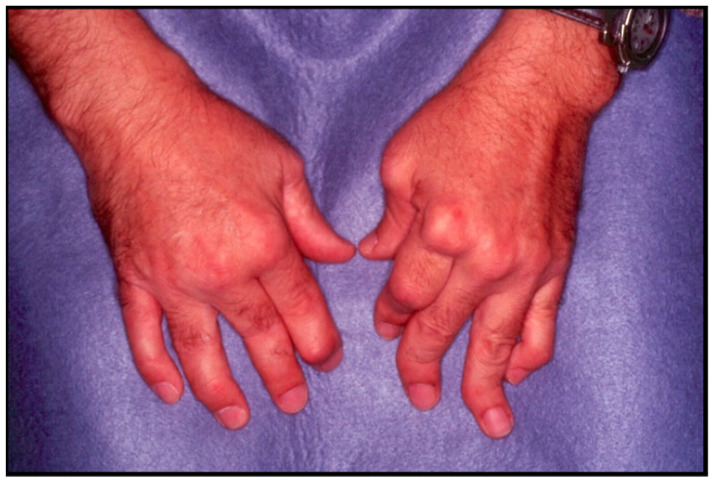
Arthritis mutilans with digital shortening.

**Figure 10:**
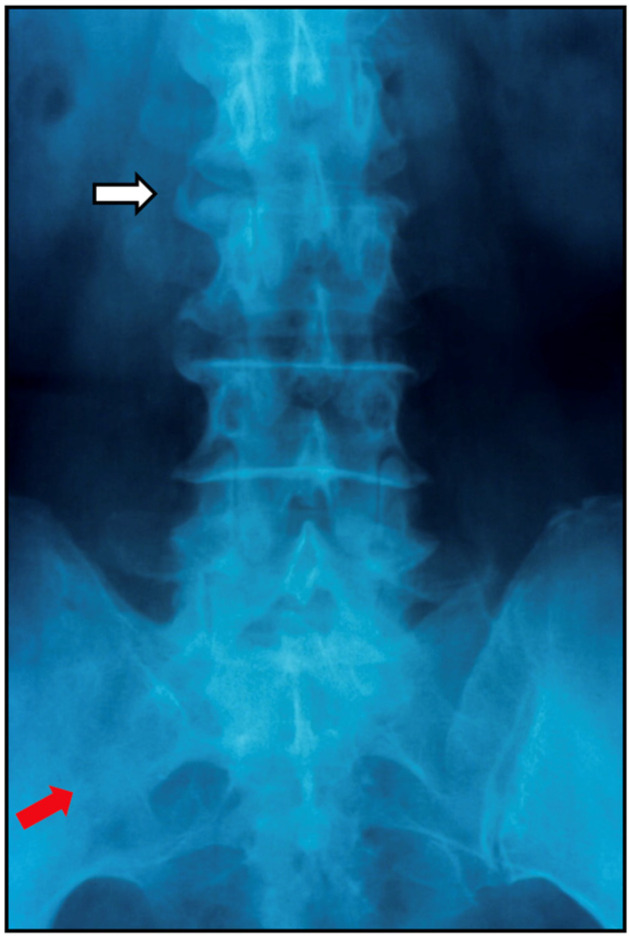
Asymmetric sacroiliitis (red arrow) and non-marginal syndesmophytes (white arrow) of axial PsA.

The clinical presentation and disease course of PsA are highly variable, making early diagnosis and treatment decisions challenging. Patients initially present with skin and nail lesions as PsO usually precedes PsA [27]. However, in some patients, the cutaneous and joint manifestations occur simultaneously, and in other patients, the joint manifestations predate the cutaneous manifestations [11]. These lesions include skin PsO (Figure 4) and nail PsO (Figure 5). Moll and Wright have categorized the musculoskeletal features into five subtypes: oligoarticular, polyarticular, distal, arthritis mutilans, and axial [28]. Patients may have both peripheral and axial involvement. Oligoarticular PsA, defined as disease limited to ≤4 joints, is the most common form on presentation, affecting approximately 60% of patients, and usually manifests in an asymmetric pattern as seen in Figure 6 [15]. Oligoarticular PsA commonly progresses to polyarticular PsA when >4 joints are affected [29]. The polyarticular subtype often affects the joints in a symmetric pattern and presents similarly to rheumatoid arthritis, as seen in Figure 7 [27]. About 5% of patients fall under the distal subtype with joint involvement predominantly in the distal interphalangeal joints; this can be seen in Figure 8 [28]. Arthritis mutilans, the most severe subtype, manifests as severe bone resorption with subsequent development of flail joints, digital shortening, and telescoping [30]. Figure 9 demonstrates an example of arthritis mutilans. The axial subtype involves the sacroiliac joints and spine and occurs in 25–70% of patients [31]. Like ankylosing spondylitis, axial PsA is a part of the spectrum of spondylarthritis. However, it differs in that it presents at an older age of onset with less frequent inflammatory back pain and distinct radiographic features of asymmetric sacroiliitis, asymmetric non-marginal syndesmophytes, defined as bony outgrowths from spinous ligaments (Figure 10), and early involvement of the cervical spine, including the facet joints [32].

**Figure 11:**
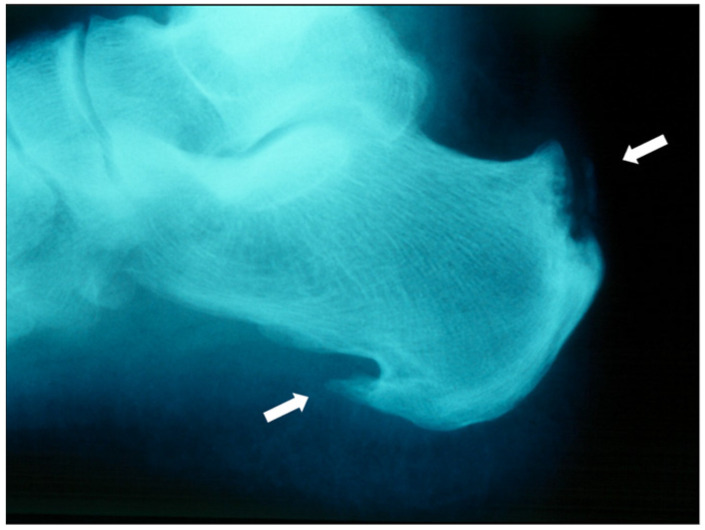
Enthesitis with erosions at the Achilles insertion (white arrows) and productive bone changes at the plantar fascia insertion.

**Figure 12:**
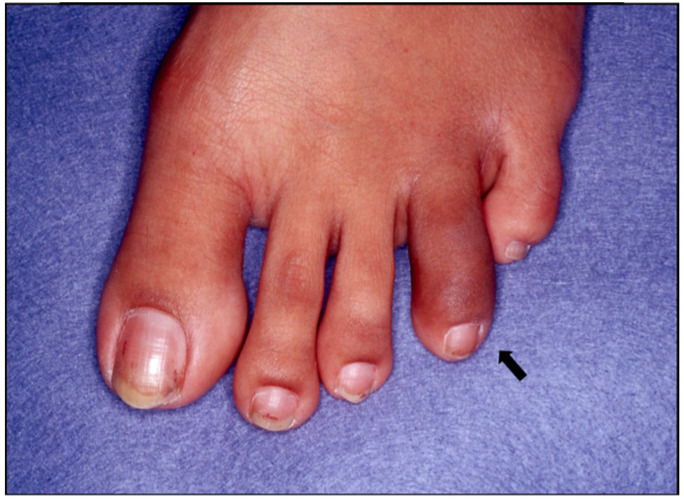
Dactylitis with diffuse swelling of 4th digit (black arrow).

Enthesitis, seen radiographically in Figure 11, and dactylitis, seen in Figure 12, are other peripheral manifestations observed in 30–50% and 40–50% of patients, respectively [33,34]. A cross-sectional analysis suggests that dactylitis is associated with greater disease burden, as patients with dactylitis have increased swollen joint count, CRP, and evidence of synovitis and erosive damage on ultrasound [35]. Uveitis is the most frequent extra-articular manifestation with a prevalence of 1.5–25% [36]. A case series in Madrid, Spain, showed that unilateral anterior acute uveitis is the most common form with a recurrent course and frequent complications of elevated intraocular pressure and cataracts [36].

## 6. Classification Criteria

There is a need to employ universal standards for PsA classification in the current era of implementation of effective biological agents. The original classification criteria were developed by Moll and Wright, followed by several others [28]. These include criteria proposed by Bennett, Gladman et al., Vasey and Espinoza, the European Spondyloarthropathy Study Group (ESSG), McGonagle et al., and Fournie et al. [11,37,38,39,40,41]. However, none of the aforementioned criteria have been universally accepted [42]. In response, the CASPAR criteria (Classification Criteria for Psoriatic Arthritis) were derived from a large prospective international study [43]. In this review, we will focus on the Moll and Wright criteria as well as the CASPAR criteria.

The original diagnostic criteria of Moll and Wright were proposed in 1973 and helped identify PsA as a separate disease entity [28]. The key components of these criteria are as follows: an inflammatory arthritis, the presence or history of psoriasis, and the absence of serological tests for rheumatoid factor [28]. Inflammatory arthritis includes arthritis of peripheral joints and/or axial involvement [28]. Using these diagnostic criteria, Moll and Wright described five subgroups of PsA: asymmetrical oligoarthritis, polyarthritis, DIP joint only, arthritis mutilans, and spondylitis [42]. The sensitivity of identifying and classifying early PsA using the Moll and Wright criteria was 80.2% in a comparative study performed by Coates in 2012, with a specificity of 99.1% [44].

The CASPAR criteria consist of confirmed inflammatory articular disease in either the joints, spine, or entheseal site, along with three points from the following features: evidence of psoriasis, defined as either current psoriasis, history of psoriasis, or family history of psoriasis; psoriatic nail dystrophy; a negative rheumatoid factor; dactylitis; juxtaarticular new bone formation [43]. See Table 1 for a summarized version of the CASPAR criteria. CASPAR criteria are said to be among the most validated criteria for PsA [45]. The objectivity of the criteria and ease of use make reliability high with a sensitivity of 91.4% and specificity of 98.7% [43]. Subsequent studies have confirmed these findings [46]. Overall, it has been demonstrated in various studies that the CASPAR criteria are more sensitive than the Moll and Wright criteria in classifying early PsA [44].

## 7. Screening Tools

### 7.1. Questionnaires

Questionnaires are screening methods that assess skin and joint involvement. The most frequently used questionnaires are the Psoriasis Arthritis Screening and Evaluation Questionnaire (PASE), the Toronto PsA Screen (ToPAS), and the Psoriasis Epidemiology Screening Tool (PEST) [33,47]. These were developed by the members of the Group for Research and Assessment of Psoriasis and Psoriatic Arthritis (GRAPPA) [47,48]. Husni et al. created PASE, a 15-item questionnaire that assessed symptoms and articular function [49]. Based on initial studies, it appeared to have a specificity of 80% and a sensitivity of 93% [50]. ToPAS was created in 2009 and consists of 12 questions regarding the skin, nails, and joints and was found to have a specificity of 93.1% and sensitivity of 86.8% [48]. A study from 2015 suggested that adding axial involvement to the ToPAS (ToPAS 2) questionnaire increases the sensitivity of the test as a screening tool, as axial disease is more likely to be part of the PsA spectrum [51]. This new version of the tool implemented images representing psoriatic lesions, arthritis, dactylitis, and nail involvement [51]. The PEST questionnaire consists of five simple questions (Ibrahim) and allows the physician to assess the affected joints with a specificity of 73% and sensitivity of 68% [52,53]. Examples of other tools are the early psoriatic arthritis screening questionnaire (EARP) and the Simple Psoriatic Arthritis Screening questionnaire (SiPAS) [54,55]. Please refer to Table 2 for a summary of the various screening questionnaires.

### 7.2. Imaging

#### 7.2.1. Plain Radiographs

**Figure 13:**
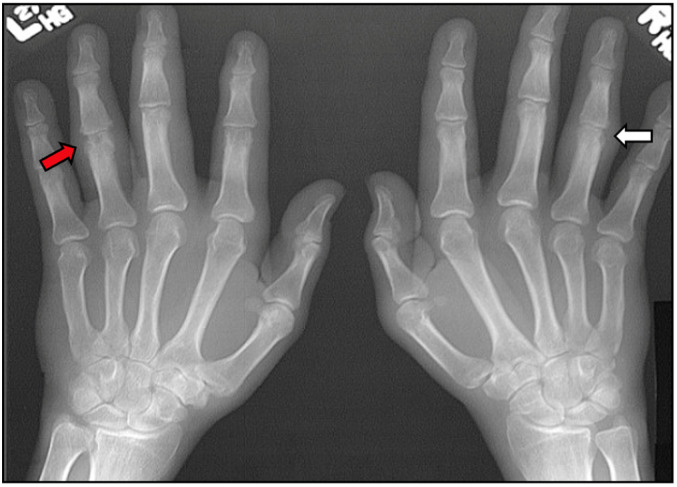
PsA with early marginal erosions (white arrow) and new bone formation (red arrow).

**Figure 14:**
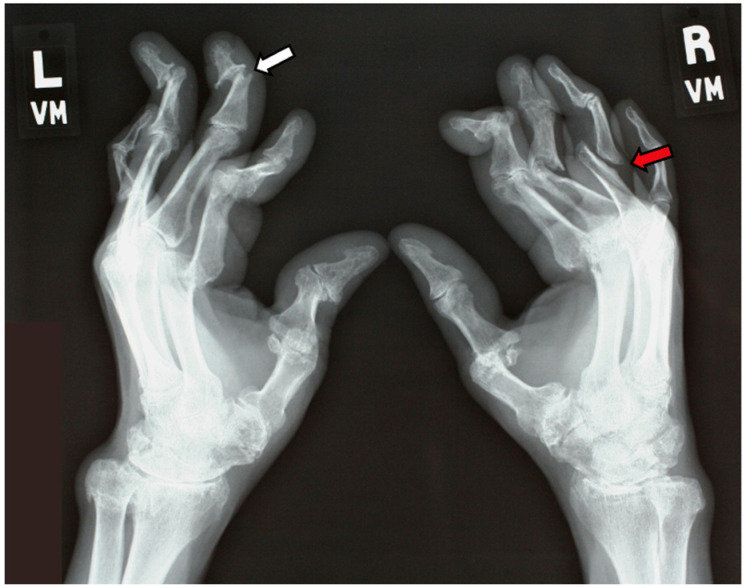
Destructive arthritis in PsA termed arthritis mutilans, “pencil-in cup” deformity (white arrow) and “whittling” (red arrow).

Plain radiographs are very useful in confirming a diagnosis of PsA. The hands, wrists, and feet are the best areas to examine with plain radiographs. Joint involvement may be symmetric or asymmetric, depending on the subtype of PsA [56]. In the hands, the metacarpophangeal, proximal interphalangeal, and distal interphalangeal can all be involved [56]. In the feet, metatarsophalangeal in interphalangeal joints may be involved [56]. Enthesitis is commonly seen in the calcaneus at the insertion of the Achilles tendon and plantar fascia with erosions and new bone formation [57]. Symmetrical joint swelling around involved joints is typical, but diffuse fusiform swelling can be seen in patients with dactylitis [57]. Juxta-articular demineralization, which is typically seen in rheumatoid arthritis, is not seen in PsA [57]. Erosion is seen in all joints of the hands, which can be marginal or central in location [56]. New bone formation, including periosteal and at entheses, is typical of PsA [56]. PsA is one of the few inflammatory arthritides that can have bone destruction (erosions) and bone formation in the same patient and even the same joint (Figure 13) [57]. Severe destructive arthritis, termed arthritis mutilans, results in severe erosive disease and deformities in the joints of the hands and feet (Figure 14) [57].

Axial disease in PsA typically has asymmetric sacroilitis, discontinuous spondylitis, and bulky non-marginal syndesmophytes [58]. (Axial disease was discussed in Section 4 and Section 5 and seen in Figure 10). Structural progression seen on plain radiograph imaging is closely linked to functional impairment and long-term disability; prevention of structural progression is, therefore, a key therapeutic goal in both clinical practice and clinical trials [57].

#### 7.2.2. Sonography

Musculoskeletal ultrasound (MSUS) has emerged as a highly valuable screening and diagnostic modality in psoriatic arthritis (PsA), particularly for detecting early or subclinical inflammatory changes in patients with psoriasis. As detailed in a systematic literature review, MSUS allows rheumatologists to objectively assess synovitis, enthesitis, dactylitis, tenosynovitis, and nail changes, lesions that often precede clinical onset and may not be visible on plain radiographs or physical examination [59,60]. Indeed, MSUS has demonstrated superior sensitivity compared to clinical exam and conventional radiography in identifying early structural and inflammatory lesions, making it a more accurate detection tool in early or atypical PsA presentations [61,62]. Importantly, in psoriasis patients without overt arthritis, subclinical ultrasound findings such as power Doppler-positive enthesitis and synovitis predict progression to clinically manifest PsA, supporting its role in proactive risk stratification [59]. Moreover, ultrasound-detected enthesopathy correlates with future radiographic joint damage and more severe disease phenotypes, underscoring its prognostic utility [59]. EULAR imaging recommendations now incorporate MSUS as a recommended adjunct in evaluating suspected or established PsA, and expert-derived algorithms have been proposed to integrate ultrasound into pragmatic clinical workflows across diagnostic and management phases [60]. Given its accessibility, cost-effectiveness, and capacity for real-time evaluation, MSUS serves as a rheumatology equivalent of the “stethoscope” in PsA, enhancing both early detection and personalized treatment monitoring in practice [62].

#### 7.2.3. MRI

Magnetic resonance imaging (MRI) provides an exceptionally sensitive method for detecting both inflammatory and structural changes in psoriatic arthritis (PsA), including synovitis, tenosynovitis, bone edema (osteitis), enthesitis, erosions, and periarticular bone proliferation, many of which are undetectable on conventional radiographs or clinical exam [61]. The OMERACT—validated Psoriatic Arthritis MRI Score (PsAMRIS) enables standardized quantification of such features—scoring synovitis, tenosynovitis, bone edema, bone erosion, bone proliferation, and periarticular inflammation—demonstrating good responsiveness to change in clinical trials and routine practice alike [61]. In patients with psoriasis but no arthritis symptoms, MRI-detected subclinical inflammation (e.g., synovitis and bone edema) combined with arthralgia significantly increases the risk of progression to PsA, with such findings associated with a substantially elevated conversion rate of more than 50% within one year [61,63]. In clinical cohorts, MRI altered treatment decisions in over half of cases when patients had suspected inflammation despite non-contributory radiographs or equivocal physical exams [64]. Moreover, MRI-detected bone edema and enthesitis are linked with more aggressive disease and radiographic progression, supporting MRI’s potential prognostic value in guiding early intervention [61]. While limitations such as availability, cost, and lack of consensus on optimal joint selection temper routine use, current EULAR and OMERACT guidance acknowledges MRI as a valuable adjunct in ambiguous cases and clinical trials, especially for early or axial PsA evaluation [63].

### 7.3. Biomarkers

Emerging evidence suggests that a range of serum and proteomic biomarkers may help predict which individuals with PsO are at risk of progressing to PsA. Elevated baseline levels of CXCL10, a chemokine induced by interferon-γ, have been strongly associated with conversion from PsO to clinically manifest PsA, with longitudinal studies demonstrating significantly higher levels in converters compared to non-converters, and each 100 pg/mL increase corresponding to roughly a 30% higher risk (OR 1.3; 95% CI 1.1–1.5; *p* = 0.004) [65]. In pilot and case–control studies, higher concentrations of hs-CRP, osteoprotegerin (OPG), MMP-3, and an increased CPII:C2C collagen degradation ratio have independently differentiated PsA patients from those with PsO alone (*p* < 0.03) [66]. Further proteomic analyses identified integrin β5 (ITGβ5) and mac-2 binding protein (M2BP) as significantly elevated in PsA versus PsO without arthritis, with adjusted odds ratios of ~3.8 and ~32, respectively, suggesting potential utility as part of a predictive panel [65]. Additional candidate biomarkers under investigation include panels involving CRP, SPP1, SOST, LEP, DEFA1, TFCP2, and CPII, particularly when combined with clinical features such as nail psoriasis, to enhance discrimination between PsO and PsA [65]. Soluble inflammatory mediators, including IL-6, IL-23, and TNF-α, along with adipokines and bone/cartilage turnover markers like C- and N-terminal telopeptides of type I collagen (CTX and NTX), have also been implicated in early PsA pathogenesis and show promise in risk stratification studies [67,68]. Though exploratory metabolomics studies have identified biochemical metabolites such as tryptophan and sphingomyelins that correlate with disease activity, their predictive role remains to be validated in prospective cohorts [69]. Taken together, these emerging data underscore the potential for multi-marker panels ideally integrated with clinical predictors to better identify psoriatic patients at high risk for developing PsA, though large-scale validation studies are still urgently needed [65,68].

## 8. Management

Prior to the year 2000, the pharmacological treatment options for PsA were very limited, as there were very few randomized therapeutic trials that specifically investigated PsA [21]. The treatment options included several conventional synthetic disease-modifying anti-rheumatic drugs (csDMARDs). However, over the past 25 years, the management of PsA has been revolutionized by the development of several biological DMARDs (bDMARDs) and targeted synthetic DMARDs (tsDMARDs) [21]. The European League Against Rheumatism (EULAR) and Group for Research and Assessment of Psoriasis and Psoriatic Arthritis (GRAPPA) have guidelines that recommend csDMARDs as first-line treatment [5]. This is then followed by bDMARDs such as TNF inhibitors (TNFi), IL-17 inhibitors (IL-17i), IL-12/IL-23 inhibitors (IL-12/23i), and IL-23 inhibitors (Il-23i) and subsequently followed by tsDMARDs, including a phosphodiesterase-4 inhibitor (PDE4i) and Janus kinase inhibitors (JAKi) [70]. In this section, the pharmacological treatment of PsA is summarized. Of note, there are several non-pharmacologic therapies, such as physical and occupational therapy, psychotherapy, and dietary approaches, that can be adjunct pharmacologic therapies [21]. See Table 3 for a comprehensive overview of PsA treatment options mapped to disease domains and grade level of evidence. Evidence grading is divided into three categories: strong, indicating multiple randomized controlled trials and guideline recommendations; moderate, indicating randomized data existing but less robust with conditional and/or second-line guideline recommendations; and limited, indicating limited or negative data for that domain [5,71].

### 8.1. NSAIDs and Glucocorticoids

Short-course NSAIDs are frequently used for symptomatic improvement of pain associated with arthritis; however, this tends to be based on anecdotal information [21]. One 12-week randomized-controlled trial investigating the efficacy of celecoxib in PsA did not demonstrate any clinical improvement of symptoms with celecoxib over placebo [72]. Nonetheless, short courses of NSAIDs, for no more than 3 months, can be utilized for symptomatic relief [73]. Although topical corticosteroids are often first-line treatment in psoriatic skin lesions, systemic steroids are generally not recommended except very selectively, as there is limited efficacy and elevated comorbidity risks [73]. If needed, oral corticosteroids should be used at the lowest possible dose for the shortest period of time [73].

### 8.2. Conventional Synthetic Disease-Modifying Antirheumatic Drugs (csDMARDs)

#### 8.2.1. Methotrexate

Methotrexate is one of the most widely used medications for PsA and has been an important therapy for over 40 years [21]. In the methotrexate in Psoriatic Arthritis trial published in 2012, subset analysis demonstrated that MTX was effective in patients with the polyarticular subset of PsA with elevated acute phase reactants [74]. MTX performed well in the SEAM-PsA trial in which it achieved nearly equivalent responses in the articular, entheseal, and skin when compared to those who received TNFi [75]. Studies have shown that for PsA, doses of MTX 15mg/week or higher are more effective than lower doses [76]. Side effects include nausea, diarrhea, and oral ulcers [76]. MTX can also be teratogenic and is important to discuss in patients of reproductive age [76]. It is important to monitor laboratory values such as liver function tests and blood counts [76].

#### 8.2.2. Sulfasalazine

Sulfasalazine is an oral medication that has efficacy in arthritis; however, an RCT demonstrated that there was no significant clinical benefit in psoriasis [77]. Side effects include gastrointestinal (nausea, vomiting, diarrhea) and laboratory abnormalities; therefore, blood counts and liver function tests should be monitored at regular intervals [77].

#### 8.2.3. Leflunomide

Leflunomide, an oral pyrimidine synthesis inhibitor, has demonstrated efficacy in managing the articular manifestations of PsA, although its effectiveness in treating skin disease is more limited [78]. Laboratory monitoring for liver function tests and blood counts is important [78].

#### 8.2.4. Cyclosporine

Cyclosporine is a calcineurin inhibitor that is frequently utilized in the management of PsO due to its strong efficacy for skin involvement [79]. Its impact on joint manifestations, however, is more modest [79]. Monitoring for renal toxicity is required [79].

### 8.3. Biological Disease-Modifying Anti-Rheumatic Drugs (bDMARDs)

#### 8.3.1. Tumor Necrosis Factor Inhibitors (TNFi)

TNF promotes inflammation in skin and joints [80]. TNFi has demonstrated efficacy across all domains of PsA and has been shown to reduce radiographic progression, making it the first-line biologic therapy recommended by most treatment guidelines for PsA [80]. The five TNFi with demonstrated efficacy in PsA when compared to placebo are: etanercept (ETN), infliximab (IFX), adalimumab (ADA), and golimumab (GOL), certolizumab pegol (CZP) [81]. The first evidence of the benefit of TNFi in PsA came from a trial that showed the effectiveness of ETN in both articular and psoriasis domains [82]. Another study established that IFX therapy helped in the articular domain by slowing progression of radiographic damage to joints as well as the psoriasis domain and overall improvement in physical function [83]. A meta-analysis showed no differences in the ACR20 response between ADA, ETN, and IFX [84]. Of note, ETN is less effective when compared to IFX, ADA, GOL, and CZP in the treatment of non-musculoskeletal manifestations associated with PsA, such as uveitis or IBD [85,86]. The use of concomitant MTX with TNFi does not improve outcomes in clinical trials and observational studies [87,88,89].

TNFi increases the risk of bacterial infections and increases the risk of reactivation of latent tuberculosis (TB) and systemic fungal infections [84,90]. Patients who are also on prednisone have an increased risk of TB and fungal infections [91]. Monoclonal antibody TNFi (IFX, ADA, GOL, and CZP) have a higher risk of TB than ETN, a receptor-based therapy [92]. There appears to be an increase in non-melanotic skin cancer, but there is no evidence of an increase in other types of solid tumors and hematologic malignancies. One study from Denmark (the DANBIO Registry) showed there was no increased risk of cancer overall, but did suggest an increased risk of colon and ovarian cancer; however, this needs confirmation [93]. Screening for latent TB and hepatitis B is recommended prior to initiation of all TNFi [84].

#### 8.3.2. IL-17 Inhibitors

IL-17 is produced in large part by CD4+ T17 (Th17) cells but also by CD8+ T17 cells, γδ T cells, NK cells, and type 3 innate lymphoid cells (ILC3). IL-17 plays a key role in preserving barrier function in the gut and integrity of the epithelium [94]. IL-17 is a broad family of related cytokines; IL-17A and IL-17F are often involved in inflammatory diseases [21]. Secukinumab (SEC) is a fully human monoclonal antibody that targets IL-17A [94]. SEC has shown superior efficacy compared to placebo in several disease domains such as peripheral arthritis, spondylitis, dactylitis, enthesitis, and skin and nail disease [95,96,97,98,99]. However, in a clinical trial where they compared SEC to TNFi head-to-head, SEC was not superior to TNFi in the musculoskeletal domain but was superior in the skin domain [100]. Ixekixumab (IXE) is another monoclonal antibody that selectively targets IL-17A [80]. Similarly to the SEC, IXE has been shown to be superior to a placebo in several disease domains but again fell short in the musculoskeletal domain when compared to TNFi [101,102]. Bimekizumab (BMK) is a monoclonal antibody that inhibits IL-17A and IL-17F with the thought that this dual inhibition would provide better outcomes than IL-17A blockade alone [80]. The BE COMPLETE study demonstrated that BMK significantly improved patients’ joint swelling when compared to placebo [103]. The BE OPTIMAL randomized controlled trial compared BMK to ADA (TNFi) and placebo [104]. BMK and TNFi had similar joint responses (ACR20, 50, and 70 responses) and greater improvement of enthesitis and dactylitis when compared to placebo [104]. BMK did show numerically better skin responses than ADA [104]. IL-17i has been shown to slow rates of radiographic progression in peripheral PsA [105,106]. Almost all clinical trials of PsA include patients with peripheral arthritis. IL-17i are also approved for ankylosing spondylitis, so they are likely effective in axial involvement of PsA. Brodalumab (BRD) is a human antibody to the IL-17 receptor (IL-17R) blocking both IL-17A and IL-17F [107]. It is approved for the treatment of PsO in many countries and has been shown to have similar efficacy to the aforementioned IL-17i in the treatment of PsA [107]. It has a Black Box warning for suicidal ideation, so it is not commonly used in clinical practice. IL-17 inhibitors are indicated for all domains of PsA involvement, except when there is associated IBD [80]. Il-17i has not been shown to demonstrate efficacy when there is associated IBD and possibly may worsen the disease process [108,109,110]. The main toxicity of IL-17i is an increased risk of infection, especially mucocutaneous candidiasis.

#### 8.3.3. IL-23 Inhibitors

IL-23 is a proinflammatory cytokine involved in the pathogenesis of psoriasis, and its inhibition yields improvement in psoriatic skin disease [111,112]. Efficacy data in the arthritis, enthesitis, and dactylitis domains are robust and akin to data from RCTs of TNFi and IL-17 inhibitors [113]. Ustekinumab (UST) is a monoclonal antibody that binds to the shared p40 subunit of IL-12 and IL-23, which blocks differentiation of Th1 and Th17 cells. Its efficacy has been demonstrated in the domains of skin, peripheral arthritis, enthesitis, and dactylitis [114,115,116]. UST has been shown to reduce radiographic progression of peripheral PsA [117]. The first IL-23 inhibitor to be approved for treatment of PsA is guselkumab (GSK), a monoclonal antibody that neutralizes IL-23 by binding to the p19 subunit [21]. Studies show that patients who are treatment naïve and patients who have previously been on TNFi both have higher treatment response rates with GSK when compared to placebo [113]. Other monoclonal antibodies to the p19 subunit of IL-23 include Risankizumab (RZK) and Tildrakizumab (TLK), both of which have shown results similar to GSK and overall have positive results [118,119]. An interesting point is that GSK, RZK, and UST do not show efficacy in axial spondyloarthropathies (axSpA) [120,121]. This finding is unexpected, given that IL-17 inhibitors have shown effectiveness in the treatment of axSpA [122]. In a post hoc analysis of the DISCOVER-2 study of GSK in PsA, patients with axial involvement did show meaningful and durable improvement in this 2-year study [119]. Overall, IL-23 inhibitors have generally good safety profiles without much increased risk [80].

#### 8.3.4. Abatacept

This is a CTLA4-Ig fusion protein that binds to CD80/CD86 on antigen-presenting cells, ultimately preventing interaction with CD28 on T cells [21]. Abatacept is usually reserved for patients who have failed treatment with TNFi; a phase III trial demonstrated that patients who had failed TNFi had modest benefit in both arthritis and psoriasis domains with abatacept [123]. Common adverse effects include gastrointestinal issues (nausea, diarrhea), upper respiratory infections, and headache [123]. Abatacept has only modest efficacy, so it is not a commonly use therapy for PsA.

### 8.4. Targeted Disease-Modifying Anti-Rheumatic Drugs (tsDMARDs)

#### 8.4.1. Phosphodiesterase 4 (PDE4) Inhibitors

PDE4 inhibition promotes an increase in cyclic adenosine monophosphate (cAMP) within cells, which prevents the synthesis of TNF, IL-23, and other proinflammatory cytokines while also elevating anti-inflammatory cytokines such as IL-10 [124,125,126]. Apremilast is an oral PDE4 inhibitor [21]. The PALACE trials have demonstrated the efficacy of apremilast in PsA; patients treated with both doses of apremilast, 20 mg or 30 mg twice daily, achieved the endpoints of the study significantly better than placebo [127,128,129,130]. The most common adverse effects of PDE4 inhibitors include GI side effects of diarrhea and nausea, as well as respiratory tract infections. Depression is also a concern, so patients should be screened before initiation of Apremilast therapy [80]. Guidelines suggest its use for patients with PsA that has skin and nail involvement, peripheral arthritis, enthesitis, and dactylitis [21]. Apremilast has modest efficacy in PsA and PsO, so it is usually used in patients with mild to moderate disease.

#### 8.4.2. Janus Kinase Inhibitors

There are four JAK molecules, JAK1, JAK2, JAK3, and tyrosine protein kinase 2 (TYK 2) [80]. The JAK molecules interact with transcription activators known as STAT to form the JAK-STAT kinase signaling system within cells [131]. This system plays a role in cytokines activating cells that are involved in PsA pathogenesis [131]. The first JAK inhibitor to be approved for treatment of PsA was tofacitinib (TOF); this inhibits JAK1 and JAK3 [21]. Phase III trials have shown efficacy of TOF when compared to placebo in treatment naïve patients as well as patients who have failed TNFi [132,133]. Other JAK inhibitors include the selective JAK1 inhibitors, Upadacitinib (UPA) and Filgotinib, the TYK2 inhibitor, deucravacitinib, and the dual JAK1 and TYK2 inhibitor Brepocitinib (BRE) [21]. In one study, UPA 15 and 30 mg were compared to ADA and placebo in patients with PsO [134]. The response to UPA 15 mg was similar to ADA and superior to placebo, while the 30 mg dose was superior to ADA [134]. The FDA-approved dose of UPA for PsA is 15 mg daily. In patients who had an inadequate response to at least one bDMARD, UPA was effective for psoriatic joint and skin disease [135]. In a post-analysis of the SELECT-PsA-1 and -2 studies, UPA was effective for patients with PsA and axial involvement [136]. Deucravacitinib is approved for PsO, and there is data on its efficacy in PsA [137]. The ORAL Surveillance study, performed exclusively with patients who had rheumatoid arthritis, found that those treated with TOF had an increased risk of cardiovascular events when compared to those treated with TNFi; though this is important and relevant data, caution must be utilized with direct extrapolation of data, as this study did not examine patients with PsA [138]. JAKi should not be considered as a first-line therapy for those over 65 years of age and those with cardiovascular risk factors such as smoking, CAD, or a history of thromboembolic events [138].

### 8.5. Preventing PsA in Patients with PsO

There have been a number of studies showing that the treatment of PsO reduces the risk of developing PsA in the future. In a retrospective, longitudinal cohort study, MTX was found to reduce the risk of future PsA in patients [139]. The overall risk of PsA in patients with PsO was 3.51 per 100 patient-years and 4.45 for the control group (no DMARD therapy) compared to 1.07 for patients treated with early MTX; Hazard Ratio (HR): 0.24 (*p* < 0.001) [139]. There have also been cohort studies showing that patients with PsO treated with bDMARDs also have a reduced risk of developing PsA over time, including peripheral and axial arthritis [17,140]. Interestingly, patients with peripheral and axial PsA who were treated with UPA had a lower risk of developing uveitis [141]. However, the retrospective nature of the design introduces inherent biases, and the focus on patients from a tertiary referral center may limit the generalizability of our findings [139]. Another limitation of our study is the lack of data regarding the specific manifestations of PsA, such as peripheral versus axial involvement.

## 9. Future Directions

The future of PsA management is moving toward integrating clinical phenotyping with biomarker-driven strategies to optimize therapy selection and predict disease trajectory. Multimodal biomarker panels incorporating inflammatory mediators (e.g., CXCL10, IL-23, TNF-α), bone and cartilage turnover products, and genetic risk variants are under active investigation for their potential to identify psoriasis patients at highest risk for PsA development and to guide personalized treatment [65,66,68]. Advances in imaging, particularly high-resolution ultrasound and MRI with standardized scoring (e.g., PsAMRIS), enable detection of subclinical synovitis and enthesitis, allowing earlier intervention before irreversible structural damage occurs [59,61].

On the therapeutic front, several novel agents are in various phases of development, aiming to expand domain-specific efficacy and address refractory disease [142,143]. These include next-generation IL-17 inhibitors such as izokibep, a small protein Affibody that inhibits IL-17A by binding to it with high affinity, with high tissue penetration, and dual IL-17A/F antagonists like the nanobody, sonelokimab, which may offer superior control of musculoskeletal and skin disease [144,145]. An oral IL-17 inhibitor, DC-806, which inhibits IL-17 signaling, is in early phase studies for PsO. An oral IL-23R antagonist, JNJ-77242113, showed efficacy for PsO [146]. In addition, selective TYK2 inhibitors (e.g., deucravacitinib, brepocitinib, and TAK-279/zasocitinib) are being evaluated for efficacy across peripheral and axial domains with favorable safety profiles compared to broader JAK inhibition [147,148,149]. Agents targeting novel pathways, such as granulocyte–macrophage colony-stimulating factor (GM-CSF) blockade (e.g., namilumab) and RORγt inhibition, are also under early clinical investigation [150,151].

Equally important will be comprehensive strategies to address comorbidities, including cardiovascular disease, metabolic syndrome, mental health disorders, and inflammatory bowel disease through coordinated multidisciplinary care [18,70]. Finally, integration of holistic and lifestyle-based interventions such as exercise, weight optimization, smoking cessation, stress reduction, and dietary modification into standard care has the potential to improve both systemic inflammation and patient-reported outcomes. Together, these innovations foreshadow a more personalized, preventative, and multidisciplinary model of PsA care, aiming not only for disease control but also for restoration of long-term health and quality of life.

## 10. Conclusions

PsA is a multifaceted inflammatory disease with highly variable clinical presentations and a significant burden on quality of life. Early identification and intervention are critical to prevent irreversible joint damage and long-term disability. This review underscores the evolving understanding of PsA’s complex pathogenesis involving genetic, immunologic, and environmental factors, as well as the expanding diagnostic and screening armamentarium, including validated questionnaires, ultrasound, MRI, and emerging biomarkers to detect PsA in its earliest stages. Dermatologists and primary care providers play a vital role in recognizing early signs and initiating timely referral to rheumatology. The therapeutic landscape has evolved dramatically over the past two decades, with conventional, biologic, and targeted synthetic DMARDs offering robust efficacy across disease domains. As research advances, a precision medicine approach leveraging clinical phenotyping, imaging, and biomarker-driven risk stratification holds promise for more individualized and effective care. Continued collaboration across specialties and integration of novel diagnostic tools will be essential in improving outcomes for patients living with PsA.

## Figures and Tables

**Table 1:** Classification Criteria for Psoriatic Arthritis (CASPAR) [43].

Inflammatory Articular Disease (Joint, Spine, or Entheseal) with ≥3 of the Following:		
Evidence of PsO (one of a, b, or c)	a Current PsO	Psoriasis is present as per rheumatologist or dermatologist
	b Personal history of PsO	History of psoriasis obtained from patient, family, or qualified health care professional
	c Family history of PsO	History of psoriasis in a first- or second-degree relative according to the patient
2. Psoriatic Nail Dystrophy	Psoriatic nail dystrophy, including onycholysis, pitting, and hyperkeratosis observed	
3. A negative rheumatoid factor	By any method except latex, but preferably by ELISA or nephelometry, according to the local laboratory reference range and negative CCP	
4. Dactylitis (one a. or b.)	a Current swelling of entire digit b History of dactylitis recorded by a rheumatologist	
5. Radiological evidence of juxta-articular new bone formation	Ill-defined ossification near joint margins (but excluding osteophyte formation) on plain X-rays of hands or feet	

**Table 2:** PsA Questionnaires ^1^.

Questionnaire Name	Description	Scoring	Sensitivity (%)/Specificity (%)
PASE	15 questions divided into 2 subscales: 7 questions that assess symptoms and 8 questions that assess function	Each question is scored from 1 to 5; Max score of 75	93%/80% (Dominguez)
ToPAS	12 questions regarding skin, nail, and joint involvement	Each question is 1 point; cut off score of 8	93.1%/86.8% (Gladman)
ToPAS 2	13 questions with focus on axial and enthesitis domains	Each question is 1 point; cut off score of 7 or 8	92.0%/77.2% (Tom)
PEST	5 yes/no items designed for easy use in dermatology or primary care	Score out of 5; cut-off ≥3 suggests PsA referral	68%/73% (Iragorri)
EARP	10-item questionnaire optimized for early PsA detection in dermatology settings	Score ≥3 suggests PsA	91%/88% (Mishra)
SiPAS	5 yes/no items for rapid dermatology use	Score ≥3 suggests PsA referral	79%/87% (Salaffi)

^1^ Abbreviations: PASE—Psoriatic Arthritis Screening and Evaluation; ToPAS—Toronto Psoriatic Arthritis Screening; ToPAS 2—Toronto Psoriatic Arthritis Screening 2; PEST—Psoriasis Epidemiology Screening Tool; EARP—Early Arthritis for Psoriatic Patients; SiPAS—Simple Psoriatic Arthritis Screening.

**Table 3:** PsA treatment overview ^1^ [5,71].

Drug Class (Examples)	Disease Domains				
	Peripheral Arthritis	Axial Disease	Enthesitis	Dactylitis	Skin
csDMARDs (Methotrexate, Sulfasalazine, Cyclosporine, Leflunomide)	Moderate (Methotrexate preferred first line)	Limited	Limited	Limited	Moderate
TNF inhibitors (Etanercept, Infliximab, Adalimumab, Golimumab, Certolizumab pegol)	Strong	Strong	Strong	Strong	Moderate
IL-17 inhibitors (Secukinumab, Ixekizumab, Bimekizumab, Brodalumab)	Strong	Moderate	Strong	Strong	Strong
IL-12/IL-23 inhibitors (Ustekinumab)	Moderate	Limited	Moderate	Moderate	Strong
IL-23 inhibitors (Guselkumab, Risankizumab, Tildrakizumab)	Moderate	Limited	Moderate	Moderate	Strong
CTLA-4 Ig (Abatacept)	Moderate	Limited	Limited	Limited	Limited
PDE-4 inhibitors (Apremilast)	Moderate	Limited	Limited	Limited	Moderate
JAK/STAT inhibitors (Tofacitinib, Upadacitinib, Filgotinib, Deucravacitinib, Brepocitinib)	Strong	Limited	Moderate	Moderate	Strong
Symptomatic therapies (NSAIDs, glucocorticoids)					
Non-pharmacological therapies (Physical therapy, occupational therapy, smoking cessation, weight loss, massage therapy, exercise)					

^1^ Abbreviations: csDMARDs—Conventional synthetic disease-modifying antirheumatic drugs; TNF—Tumor necrosis factor; IL—interleukin; CTLA—Cytotoxic T-Lymphocyte-Associated Protein; PDE—Phosphodiesterase; JAK—Janus Kinase; STAT—Signal transducer and activator of transcription proteins.

## Data Availability

Not applicable.
